# The Chinese

**Published:** 1892-04

**Authors:** 


### BOOK NOTICES.

THE CHINESE: Their Present and Future; Medical, Political, and Social. By Robert Coltman, Jr., M. D., Surgeon in Charge of the Presbyterian Hospital and Dispensary at Teng Chow Fu; Consulting Physician of the American Southern Baptist Mission Society; Examiner in Surgery and Diseases of the Eye for the Shantung Medical Class; Consulting Physician to the English Baptist Missions, etc. Illustrated with Fifteen Photo-Engravings of persons, places, and objects characteristic of China. In one handsome royal octavo volume of 220 pages, extra cloth. Price, $1.75, net. Philadelphia: The F. A. Davis Co., Publishers, 1231 Filbert Street.

This book reads like a novel, and once begun will not be laid aside till completed. The author has spent many years among the Chinese; lived with them in their dwellings; thoroughly learned their language; dressed in their peculiar habit; has become conversant with all their strange and odd characteristics to a greater extent than almost any other American. He has been a physician to all classes of this wonderful people, and the opportunities thus afforded for a clear insight into the inner life of the Chinese; their virtues and vices, political, social, and sanitary condition, probable destiny, and their present important position in the world to-day have been ably and wisely used by Dr. Coltman. He pictures the character of the Celestial just as we see him here in our country. As a people, they are never in a hurry, but sluggish in the extreme. Sympathy is out of question with them, and good Samaritans are even more rare than in Palestine. If half is true what Dr. Coltman writes regarding the morals of the Chinese, they well compare with the Americans or English. Adultery may even be punished with the death of the guilty parties, which in civilized countries is not possible. He saw a woman and her paramour led to execution down the principal street of Chinanfu for adultery, and the suspicion of having killed the woman's husband, and all around him the people kept saying: "A righteous verdict," "They deserve to die," and other like comments expressive of their disgust at the crime, and their satisfaction with the extreme penalty about to be administered.

One of the most interesting chapters is that upon leprosy. The author doubts the excessive contagiousness of leprosy. Thus, at page 155, he says: "Leprosy cannot be as contagious or infectious as a great many alarmists in and outside the medical profession in the United States would have us believe; for if so, the people of China would have disappeared from the face of the earth from leprosy, long ago. My reason for this statement lies in the fact that, although there is leprosy existing in every province of the empire and every city of size, yet in spite of the fact that the leper is under no quarantine regulation of any kind, leprosy has not spread to any appreciable degree in the last century. If leprosy is actively contagious, would not the leper handling money, farm implements, and even food-products, be a centre for the distribution of the disease?"

At page 161 he says: "I believe, from what I have seen, that the disease is hereditary in most cases; that it is feebly contagious; that it is inoculable. Many of my patients with leprosy have acknowledged having had syphilis, and I believe that the previous saturation of the body with syphilis affords a favorable soil for the development of the disease." * * * * "Given a proper soil, and the bacillus introduced, the specific disease is certain to follow. Introduce it upon a barren or uncongenial soil, and the death of the bacillus would follow. This proposition seems undeniable, reasoning from analogy. If it is so, the syphilitic body seems a very favorable soil for the growth of the bacillus of leprosy. The Hawaiian Islanders were a strong, hardy race, apparently, up to the time the whalers from the Pacific infected the people with syphilis, which spread until, it is reported, they were about all syphilized; then leprosy took hold, and to-day the settlement of Molokai, the largest leper settlement in the world, is the result. * * * * I don't want to be understood as saying all lepers are syphilitic. My position is, that a syphilitic person is more apt to become leprous upon exposure to contagion for a length of time than an otherwise healthy individual would be."

The above extract is particularly interesting to homœopathists. The book is full of interest to everybody, and our thanks are due to Dr. Coltman for the admirable style with which he handles his subject. W. S.

